# Butyrate Induces Modifications of the CTCF-Binding Landscape in Cattle Cells

**DOI:** 10.3390/biom12091177

**Published:** 2022-08-25

**Authors:** Clarissa Boschiero, Yahui Gao, Ransom L. Baldwin, Li Ma, Cong-jun Li, George E. Liu

**Affiliations:** 1 Animal Genomics and Improvement Laboratory, Beltsville Agricultural Research Center, Agricultural Research Service, U.S. Department of Agriculture, Beltsville, MD 20705, USA; 2 Department of Animal and Avian Sciences, University of Maryland, College Park, MD 20742, USA

**Keywords:** CTCF, cattle, butyrate, differentially CTCF-binding sites, MDBK cells

## Abstract

Butyrate is produced in the rumen from microbial fermentation and is related to several functions, including cell differentiation and proliferation. Butyrate supplementation in calves can accelerate rumen development. DNA-protein interactions, such as the CCCTC-binding factor (CTCF), play essential roles in chromatin organization and gene expression regulation. Although CTCF-binding sites have been identified recently in cattle, a deeper characterization, including differentially CTCF-binding sites (DCBS), is vital for a better understanding of butyrate’s role in the chromatin landscape. This study aimed to identify CTCF-binding regions and DCBS under a butyrate-induced condition using ChIP-seq in bovine cells; 61,915 CTCF peaks were identified in the butyrate and 51,347 in the control. From these regions, 2265 DCBS were obtained for the butyrate vs. control comparison, comprising ~90% of induced sites. Most of the butyrate DCBS were in distal intergenic regions, showing a potential role as insulators. Gene ontology enrichment showed crucial terms for the induced DCBS, mainly related to cellular proliferation, cell adhesion, and growth regulation. Interestingly, the ECM-receptor interaction pathway was observed for the induced DCBS. Motif enrichment analysis further identified transcription factors, including CTCF, BORIS, TGIF2, and ZIC3. When DCBS was integrated with RNA-seq data, putative genes were identified for the repressed DCBS, including *GATA4*. Our study revealed promising candidate genes in bovine cells by a butyrate-induced condition that might be related to the regulation of rumen development, such as integrins, keratins, and collagens. These results provide a better understanding of the function of butyrate in cattle rumen development and chromatin landscape regulation.

## 1. Introduction

Various physiological functions of the rumen are regulated by short-chain fatty acids (SCFAs), also called volatile fatty acids (VFAs), which are molecules derived from the rumen microbial fermentation. The main SCFAs produced on the rumen are acetate, propionate, and butyrate. These SCFAs can affect the rumen epithelium integrity and renewal of rumen epithelial cells [1,2]. In addition, SCFAs contribute to a substantial proportion of the energy requirement of ruminants (~70% in cattle) [3].

Butyrate is present in relatively low concentrations during rumen fermentation compared to acetate and propionate. However, it involves several important roles, such as cell differentiation, proliferation, and apoptosis, and can cause DNA histone modifications [4,5,6,7]. Previous studies investigated butyrate-induced histone modifications in cattle [8,9], but it remains largely unknown how histone modifications are regulated. Butyrate plays an important role in cattle nutrition and rumen development and is a crucial regulator of genomic activities [4,6].

Studies show that butyrate supplementation in young calves has beneficial effects on gastrointestinal performance and growth rates. For example, preweaning calves supplemented with butyrate develop a rumen and mature ruminal epithelium more rapidly [10]. Another study observed that butyrate supplementation in dairy calves resulted in positive trends in growth rate and feed efficiency and may affect the gastrointestinal microbiota [11]. Butyrate utilization can also result in bovine immune and inflammatory responses [12]. Studies also showed that the butyrate effect is not restricted to the rumen but also helps the development of the small intestine, pancreas, and abomasum [10,13]. These studies showed that butyrate is crucial to gastrointestinal tract development.

Recently, Chromatin immunoprecipitation followed by sequencing (ChIP-seq) and Assay of Transposase Accessible Chromatin sequencing (ATAC-seq) have been used to identify regulatory elements in cattle [14,15,16,17]. The ChIP-seq approach can detect DNA-protein interactions across the genome that play essential functions in gene expression regulation and chromatin organization [18]. DNA binding proteins include histone modifications, transcription factors, and CCCTC-binding factor (CTCF). The CTCF uses 11 zinc fingers to bind the genome and is a crucial chromatin organizer in vertebrates [19]. CTCF and cohesin work cooperatively to control chromatin architecture by folding the genome into loops and domains called topologically associating domains that are crucial elements of nuclear organization [19,20]. CTCF has several fundamental roles, including regulation of the three-dimensional chromatin structure, regulation of gene expression, insulation activity by blocking the interaction between enhancers and promoters, regulation of mRNA splicing, and reparation of DNA double-strand breaks [19,21,22,23,24]. However, the mechanisms by which CTCF performs its functions remain largely unclear, and more information about this versatile protein is needed to be better understood.

Millions of bovine regulatory elements have been identified in the last years; however, identifying such regulatory elements in specific tissues and conditions is still lacking or has not been conducted in a more profound aspect. CTCF ChIP-seq studies have been conducted recently in sheep [25,26] and cattle [14,27,28]. CTCF-binding regions have been recently identified in dairy cows using ChIP-seq data from six different tissues (heart, kidney, liver, lung, mammary, and spleen) [27]. In another study, CTCF binding and chromatin accessibility were discovered in eight tissues from Hereford cattle [28]. In addition, the first global map of regulatory elements in bovine rumen epithelial cells under a butyrate treatment was generated, including CTCF-binding sites [16]. Although CTCF-binding sites were identified in these studies in cattle, a deeper characterization of the CTCF regions, including differentially CTCF-binding sites (DCBS), is crucial to better understand the butyrate roles in the cattle chromatin organization. Because of the facts presented here, the main objectives of this study were to identify and characterize CTCF-binding regions and DCBS under a butyrate-induced condition using ChIP-seq to identify cis-regulatory elements in bovine cells.

## 2. Methods

### 2.1. MDBK Cells and Butyrate Treatment

The Madin–Darby bovine kidney epithelial cells (MDBK; American Type Culture Collection, Manassas, VA, USA; Catalog No. CCL-22) were cultured in Eagle’s minimal essential medium and supplemented with 5% fetal bovine serum (Invitrogen, Carlsbad, CA, USA) in 25 cm^2^ flasks, as described in our previous report [5]. At approximately 50% confluence, the cells were treated with five mM of sodium butyrate for 24 h during the exponential phase (Calbiochem, San Diego, CA, USA). A butyrate concentration of 5 mM was selected as it represents a physiologically relevant dose and has previously been successfully used to evoke desired changes in the cell cycle dynamics [5]. One flask of cells for both butyrate treatment and the control groups (two samples in total) was used for the ChIP-sequencing experiments.

### 2.2. CTCF ChIP-seq

CTCF ChIP-seq in bovine cells from two samples (butyrate and control) was performed by Active Motif, Inc. (Carlsbad, CA, USA). The DNA integrity was verified using the Agilent Bioanalyzer 2100 (Agilent; Palo Alto, CA, USA), then the DNA was processed, including end repair, adaptor ligation, and size selection, using an Illumina sample prep kit, following the manufacturer’s instructions (Illumina, Inc., San Diego, CA, USA). The DNA libraries were sequenced (75 bp) on an Illumina HiSeq 2500 platform (Illumina, San Diego, CA, USA).

### 2.3. Sequencing Data Processing

The read quality was examined using FastQC v.0.11.9 (https://www.bioinformatics.babraham.ac.uk/projects/fastqc/, (accessed on 14 March 2022)). Then, reads were aligned to the ARS-UCD1.2 cattle reference genome assembly [29] using BWA v.0.7.17 with default settings [30]. Unmapped reads, reads mapped to multiple locations, reads located on the mitochondrial chromosome, and reads with a mapping quality (MAPQ) < 10 were removed by SAMtools v.1.9 [31]. Also, duplicate reads were removed using Picard v.2.22.3 (https://broadinstitute.github.io/picard/, (accessed on 30 March 2022)). 

### 2.4. CTCF Peak Calling

Individual CTCF peaks were identified using MACS2 v.2.2.7.1 [32] with default parameters and FDR < 0.05. Peaks located on Chromosome X and unplaced were removed. The fraction of all mapped reads in enriched peaks (FRiP) was obtained for each sample for quality check. The BEDtools v.2.25.0 [33] intersect option was used to obtain each condition’s specific number of peaks.

### 2.5. Identification of Differentially CTCF-Binding Sites

DiffReps v.1.55.6 [34] was used to identify the DCBS of the butyrate (butyrate vs. control conditions) using a G-test (*p*-value < 0.05) and a defined window of 200 bp. The DCBS were filtered with a |log_2_FC| >1 and mapped against the identified CTCF peaks. The CTCF peaks from the two conditions (butyrate and control) were merged by BEDtools v.2.25.0 [33] to generate a list of non-redundant peaks. Then, the DCBS were overlapped against the non-overlapping peaks using BEDtools v.2.25.0 [33] with intersect function. The DCBS that coincided with MACS2 peaks in at least one sample were further analyzed. The induced and repressed DCBS were separate based on their log_2_ fold change values and used for the downstream analyses.

### 2.6. Annotation of the Differentially CTCF-Binding Sites

The induced and repressed DCBS were annotated with the annotatePeak from the ChIPseeker package [35]. ChIPseeker [35] uses the nearest gene method for the peak annotation. Promoter regions were defined as ±2 kb from the transcription start site (TSS). Also, the annotatePeak function from the ChIPseeker [35] was used to plot the distance of the DCBS around the TSS. 

### 2.7. Gene Ontology Enrichment and Pathway Analysis

Gene ontology (GO) enrichment and KEGG pathways analysis were performed with ShinyGO v.0.76 [36] (FDR < 0.05) using 1561 genes annotated from the induced DCBS and 225 genes annotated from the repressed DCBS to obtain enriched biological process (BP), cellular component (CC), and molecular function (MF) terms. Also, QIAGEN Ingenuity Pathway Analysis (IPA) v.68752261 [37] was used to find signaling and metabolic pathways, including canonical pathways (*p*-value < 0.01), upstream regulators (*p*-value of overlap < 0.01), and molecular networks (network score > 20) using 1561 genes from the induced DCBS and 225 genes from the repressed DCBS. 

### 2.8. Motif Enrichment

The enriched motifs were identified using HOMER v.4.11 [38] from the induced and repressed DCBS with the findMotifsGenome function (*p*-value ≤ 0.01 and >5% of target sequences with motif). 

### 2.9. RNA-seq Integration with CTCF Data

To investigate gene expression and regulatory networks and compare with DCBS, previously RNA-seq data from butyrate and control conditions (three biological replicates for each condition totaling six samples) were utilized [16] to obtain differentially expressed genes (DEG) (data are available at the NCBI’s Gene Expression Omnibus database; accession number GSE129423). RNA-seq clean reads (Q > 20) were aligned to the ARS-UCD1.2 cattle genome [29] with STAR v.2.7 [39], and gene expressions and DEG were obtained with Cufflinks v.2.2.1 [40]. The integration of DEG and DCBS was performed with the BETA tool v.1.0.7 [41] with a *p*-value of 0.05. The analysis was performed for the induced and repressed DCBS jointly. The *p*-values from BETA (rank product) are estimated by the Kolmogorov–Smirnov test comparing the regulatory potential of upregulated, downregulated, and background genes. Genes with *p*-values/rank product < 0.05 were considered significant target genes.

### 2.10. Visualization of the CTCF Signals in Selected Genes

The bigwig files from butyrate and the control were generated from MACS2 bedGraph files using the bedGraphToBigWig tool [42]. Then, the bigwig and bed files from the induced and repressed DCBS were visualized using the Integrative Genomics Viewer (IGV) [43] for selected genes.

## 3. Results

### 3.1. Sequencing Data Quality

ChIP-seq libraries were obtained from MDBK cells treated with butyrate and the control. A total of 40,990,109 reads were initially generated for butyrate and 39,610,540 reads for the control, and approximately 90% of the reads were aligned to the ARS-UCD1.2 cattle reference genome assembly [29] with a total of 72,567,299 reads mapped (Table 1). On average, 0.02% of the reads were mapped to the mitochondrial genome, 14.5% were duplicated, and ~19% had a MAPQ score < 10. A total of 23,691,050 clean reads were produced for butyrate, and 23,673,693 for the control, respectively.

### 3.2. CTCF Peaks and Differentially CTCF-Binding Sites

**Figure 1:**
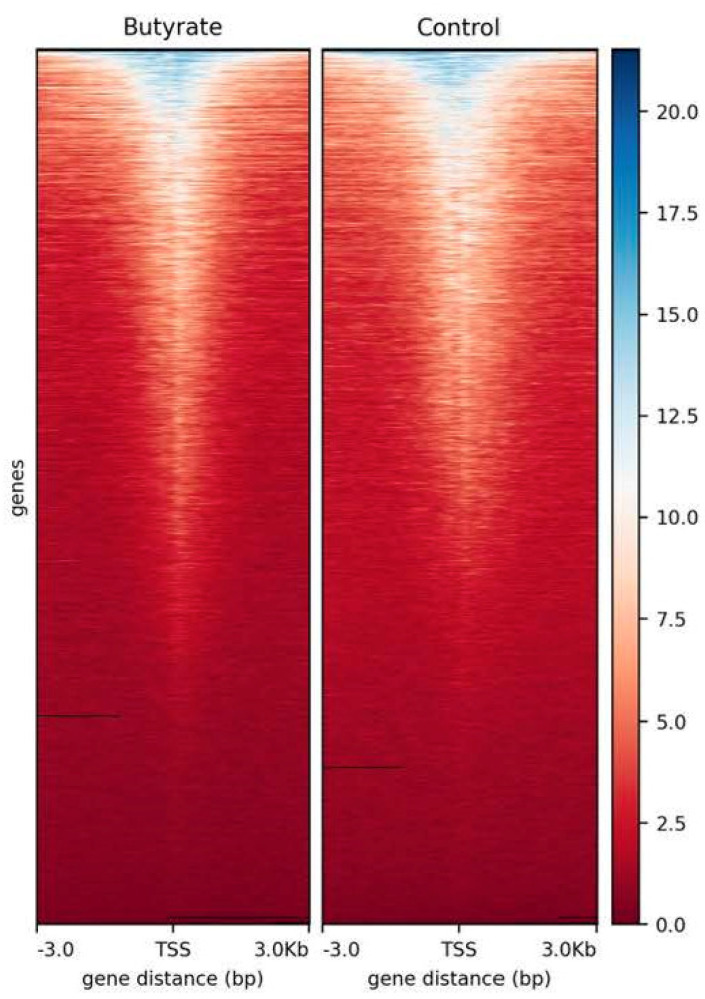
Heatmap profile of CTCF peaks relative to the transcription start sites (TSS) considering a ±3 kb region for each pool with two samples each (considering chromosomes 1–29). Blue color intensity reflects the level of peak enrichment, and black lines are missing data. BT: butyrate. CT: control.

The CTCF peaks were discovered in the individual samples by the MACS2 (FDR < 0.05) from 47,364,743 clean reads [32]. A total of 61,915 CTCF peaks were identified in the butyrate and 51,347 peaks in the control (Table 2). The average CTCF peak length was 509 for butyrate and 426 for the control. The quality of the CTCF-seq data was evaluated by calculating the fraction of reads in peaks (FRiP) of each sample. The butyrate FRiP was 0.22, and the control was 0.16. Also, the specific number of peaks in the butyrate and the control conditions was obtained, showing a total of 21,392 butyrate-specific CTCF peaks and 9234 control-specific peaks, and the consensus number of shared peaks was 42,630. In addition, the heatmap profiles of peaks relative to TSS were generated to evaluate the quality of the CTCF peaks (Figure 1).

**Figure 2:**
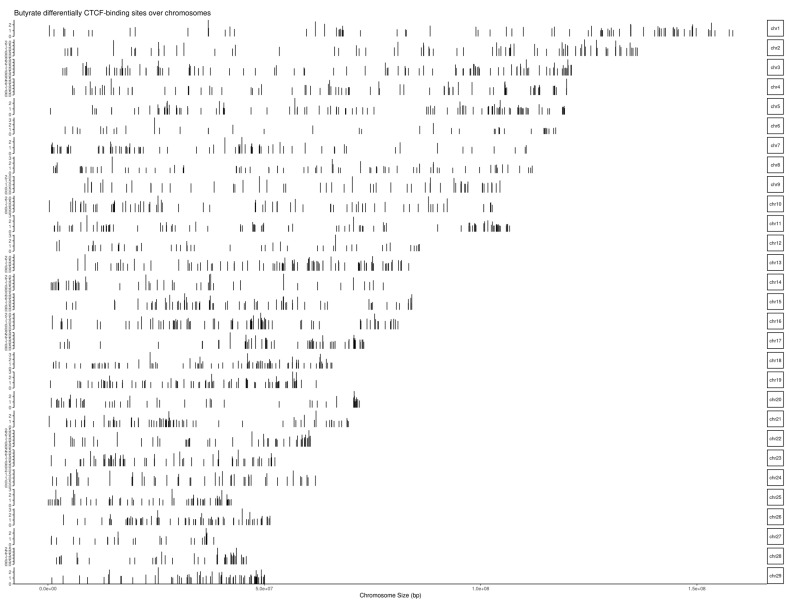
Chromosomal distribution of 2265 butyrate differentially CTCF-binding sites (considering bovine chromosomes 1–29).

After the CTCF peak calling, the DCBS were identified using Diffreps [34]. A total of 16,532 DCBS (*p*-value < 0.05) was initially obtained for the butyrate vs. the control (Table 3). The DCBS were filtered based on |log_2_FC| > 1, and approximately 14% were retained, totaling 2355 sites. Then, the 2355 sites were overlapped against a list of 70,625 CTCF merged peaks from butyrate and control conditions (Table S1), and most of the regions overlapped with peaks, totaling 2265 sites representing 0.035% of the cattle genome (Figure 2), including ~90% of induced sites (log_2_FC ≥ 1), and ~10% of repressed sites (log_2_FC ≤ −1) (Table 3 and Table S1).

**Figure 3:**
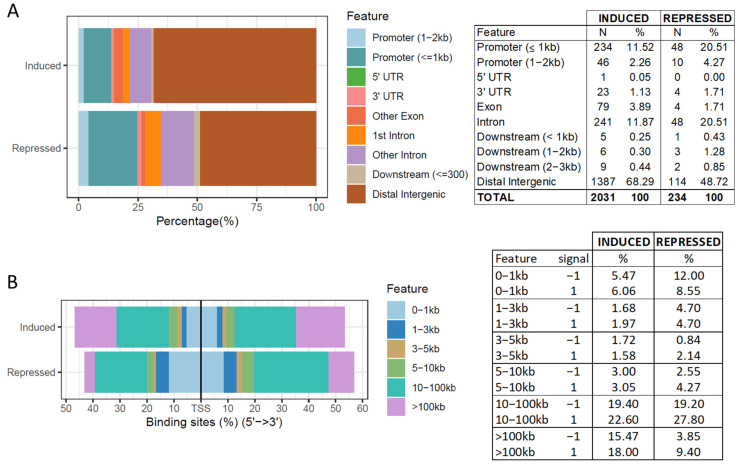
(**A**) Annotation of butyrate-induced and -repressed differentially CTCF-binding sites. (**B**) Distribution of the butyrate-induced and -repressed differentially CTCF binding sites relative to TSS.

The annotation of the 2265 butyrate-induced and -repressed DCBS was done separately (Figure 3 and Table S2). Most of the butyrate-induced and -repressed DCBS were in distal intergenic regions (~69% for the induced and 49% for the repressed), followed by promoters (~14% for the induced and 25% for the repressed), and introns (~12% for the induced and 21% for the repressed) (Figure 3A). Furthermore, the distribution of the induced and repressed sites relative to TSS showed that the majority of the induced DCBS fall in 10–100 kb and >100 kb regions around the TSS, and most of the repressed DCBS fall in 10–100 kb and 0–1 kb regions around the TSS (Figure 3B).

### 3.3. Gene Ontology Enrichment and Pathway Analysis

**Figure 4:**
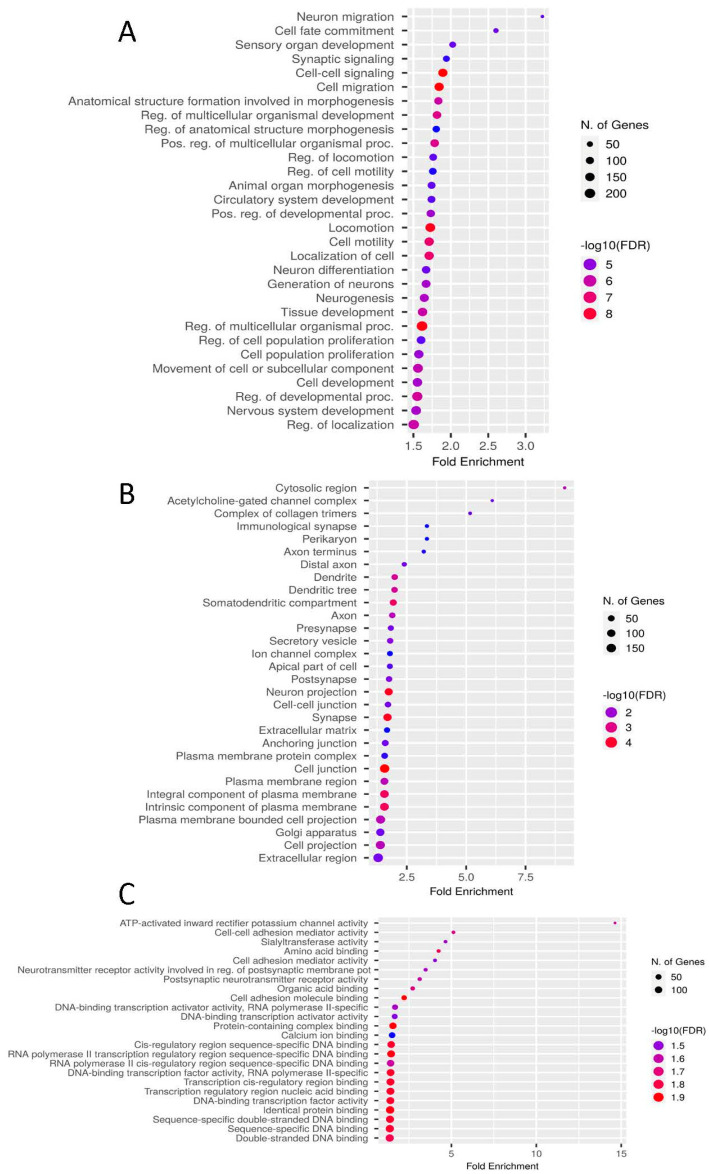
GO enrichment results for the butyrate-induced differentially CTCF-binding sites show the top GO terms for (**A**) biological process, (**B**) cellular component, and (**C**) molecular function. Biological processes are ranked according to the fold enrichment values. Bubble colors represent the *p*-value of the False Discovery Rate (FDR). The most important processes are highlighted in red, and the less important processes are highlighted in blue according to log_10_(FDR) values. Bubble sizes indicate the number of genes.

GO enrichment and KEGG pathway analysis were conducted with ShinyGO (FDR < 0.05) [36]. A total of 1561 unique genes annotated from the 2031 induced DCBS and 225 unique genes from the 234 repressed DCBS were utilized. The repressed DCBS were significantly enriched for only five BP terms—protein localization, reticulum, cellular protein localization, cellular macromolecule localization, and retrograde vesicle-mediated transport (Table S3). The induced DCBS were significantly enriched for 387 BP terms, 35 CC terms, and 24 MF terms (Table S4 and Figure 4). There are several crucial BP terms related to cell migration and motility, cell development, and epithelial cell proliferation. In addition, we identified five terms related to cell adhesion and cell junction (e.g., cell–cell adhesion, cell junction organization), five terms related to growth (e.g., regulation of growth, positive regulation of growth), nine terms related to epithelial cells proliferation or migration, and 11 terms related to the regulation of hormones for the induced regions (Table S4). Induced sites were also enriched for terms related to the cell adhesion and junction, cell projection, plasma membrane, anchoring junction, and complex collagen trimers (Table S4). Furthermore, KEGG enrichment analysis revealed five significant pathways for the induced DCBS, including calcium signaling, extracellular matrix (ECM)-receptor interaction, and metabolic pathways (Table S5).

### 3.4. IPA Pathways

IPA [37] was utilized to generate relevant biological pathways from the 1561 genes from the butyrate-induced DCBS and 225 genes from the repressed DCBS. Significant networks (network score > 20) from the induced sites were related to cell morphology, assembly, development, growth, proliferation, and signaling (Table S6). Networks from the repressed sites were related mainly to the cell cycle and movement, and organ development (Table S6). Significant canonical signaling pathways (*p*-value < 0.01) were identified for the induced DCBS, including growth hormone, macropinocytosis signaling, regulation of cellular mechanics by calpain protease, AMPK, PAK, and others (Table S7). Eight canonical pathways were identified for the repressed DCBS, including CLEAR signaling, regulation of the epithelial–mesenchymal transition, WNT/β-catenin, and others (Table S7). Significant upstream regulators (*p*-value < 0.01) such as growth factors, kinases, and transcription factors were identified for the induced sites (e.g., TGFβ1, IGF1/2, TGFBR2, MTOR, JUN, SMAD1-4, SP1/7, ITGA2), and for the repressed sites (e.g., TGFβ1-3, TGFBR1/2, JUN, FOS, SMAD1-4, SP1). More details can be found in Table S8.

### 3.5. Motif Enrichment Analysis

The HOMER tool [38] was employed to identify enriched motifs (*p*-value ≤ 0.01) and putative transcription factor binding sites (TFBS) in the butyrate-induced and repressed DCBS. Twenty-six and 41 enriched motifs were identified for the butyrate-induced and repressed DCBS, respectively (Table S9). The top ten TFBS (ranked according to *p*-values) for the induced sites were BORIS, CTCF, ZIC3, BCL11A, THRB, ZIC2, LRF, ZEB1, NEUROD1, and SCL, and for the repressed sites were CTCF, BORIS, FRA2, FOS, JUN-AP1, ATF3, FRA1, JUNB, AP1, and BATF.

### 3.6. RNA-seq Integration with CTCF ChIP-seq Data

**Figure 5:**
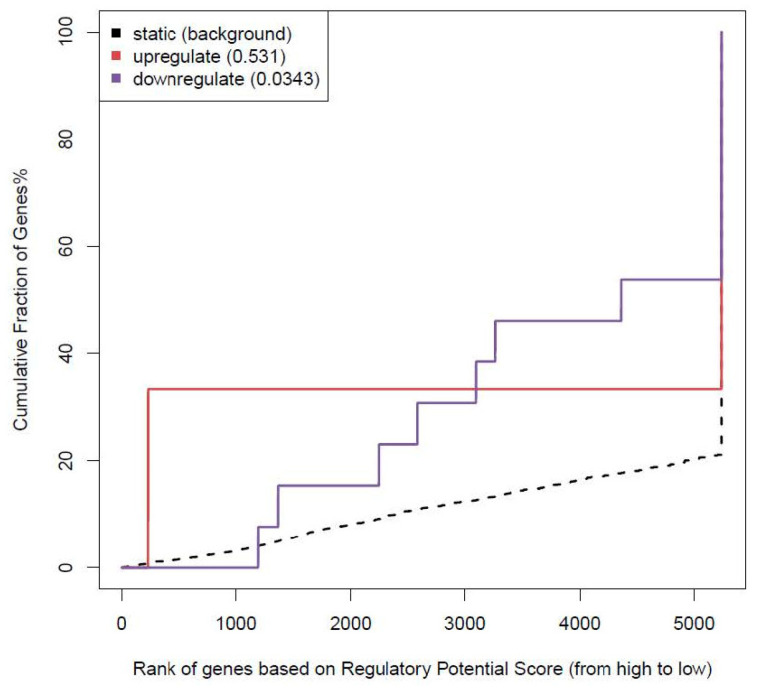
BETA activating and repressive function prediction of butyrate differentially CTCF-binding sites. The red line represents the upregulated genes, the purple line represents the downregulated genes, and the dashed line represents background genes with no differentially expressed genes. The *p*-values (inside the box) represent the significance of the difference between the up/down groups compared with the background genes by the Kolmogorov–Smirnov test.

BETA [41] was used to identify putative target genes and to predict whether the factor has an activating or repressive function by integrating ChIP-seq data with differential gene expression data. DCBS were integrated with previous RNA-seq data from butyrate [16]. The functional impact of induced and repressed DCBS was gene-repressing/downregulating (*p*-value = 0.0343, Figure 5), and seven target genes (rank value/*p*-value < 0.05) were identified, including *GATA4*, *RASD1*, *KANK3*, *CFAP45* and *ZNF395* (Table S10).

### 3.7. Visualization of CTCF Signals in Selected Genes

**Figure 6:**
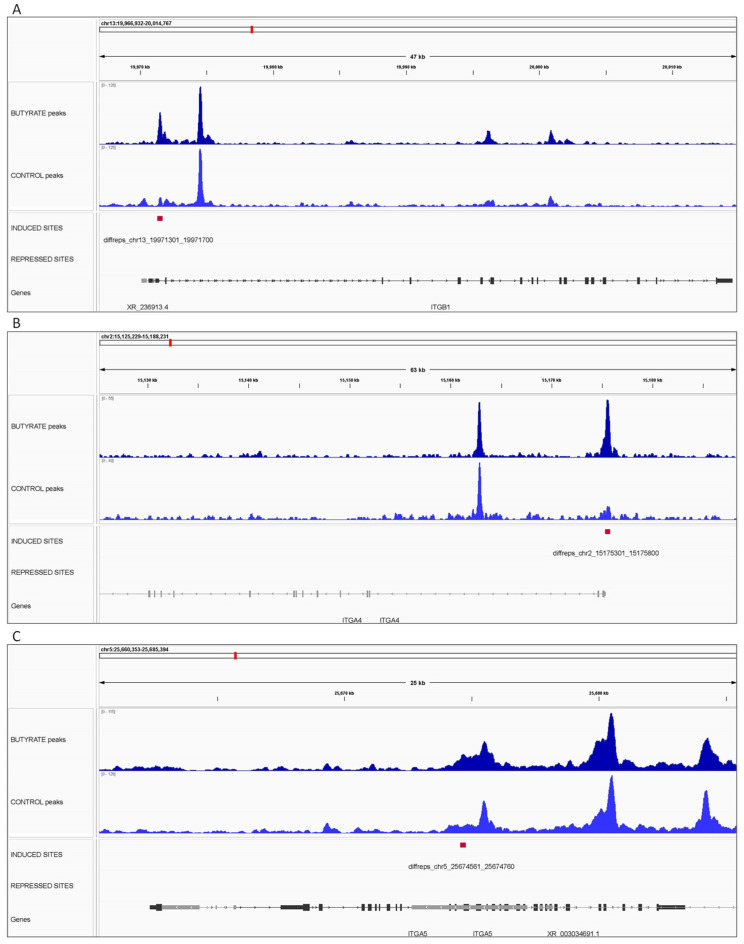
Integrative genomics viewer (IGV) screenshot of CTCF peaks (BT and CT) and the butyrate-induced and -repressed differentially CTCF-binding sites for the (**A**) *ITGB1* gene, (**B**) *ITGA4* gene, and (**C**) *ITGA5* gene. The *X*-axis represents the chromosomal location with the size bar given in kb.

**Figure 7:**
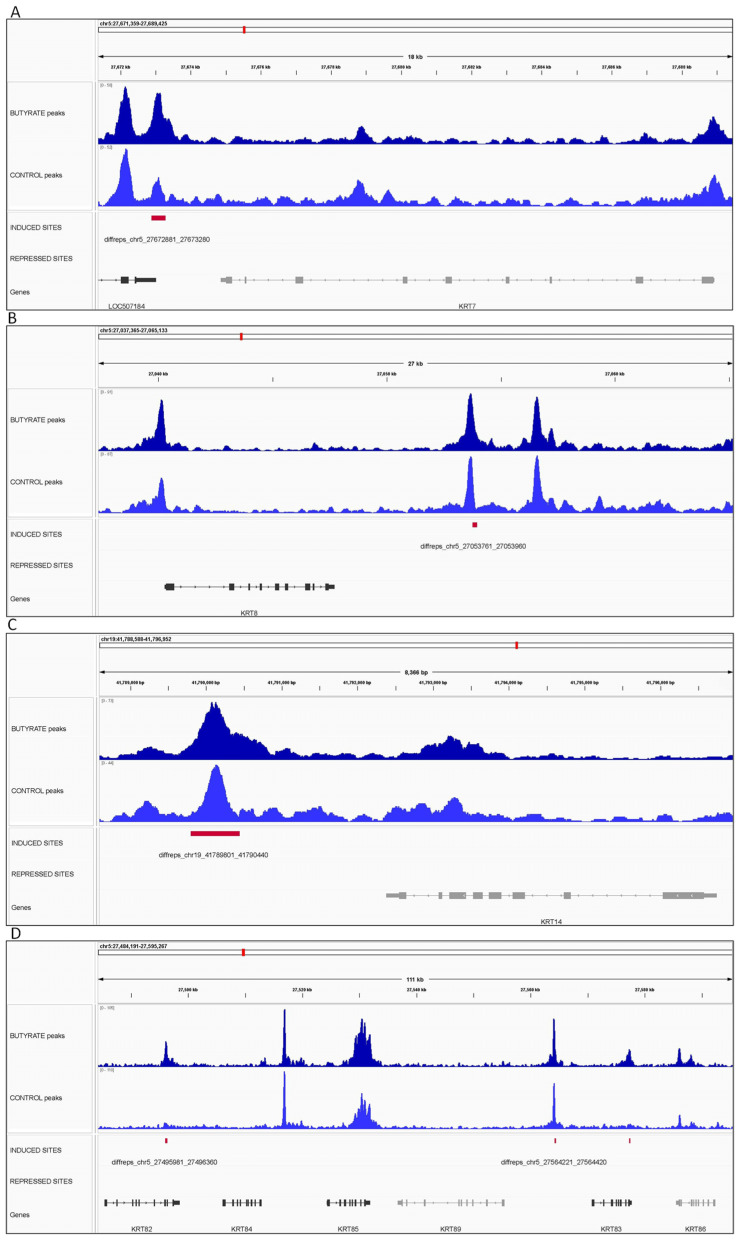
Integrative genomics viewer (IGV) screenshot of CTCF peaks (BT and CT) and the butyrate-induced and -repressed differentially CTCF-binding sites for *keratin* genes including—(**A**) *KRT7*, (**B**) *KRT8*, (**C**) *KRT14*, and (**D**) *KRT82*, *KRT83, KRT84, KRT85, KRT86*, and *KRT89* genes. The *X*-axis represents the chromosomal location with the size bar given in kb.

The CTCF peaks from butyrate and control conditions and the butyrate-induced and -repressed DCBS were examined in the IGV tool [42] for selected genes related to potential roles in rumen development during butyrate treatment, such as cellular adhesion and cell growth. The selected genes were *ITGB1, ITGA4,* and *ITGA5* (Figure 6). In addition, nine *keratin* genes were selected, including *KRT7*, *KRT8*, *KRT14*, *KRT82*, *KRT83*, *KRT84*, *KRT85*, *KRT86*, and *KRT89* (Figure 7). All the selected genes were located near butyrate-induced DCBS.

## 4. Discussion

In this study, we utilized ChIP-sequencing data from the MDBK cells with and without butyrate treatment to generate a comprehensive collection of bovine CTCF-binding sites and differentially CTCF-binding sites. We observed distinct CTCF-binding site profiles at different conditions, indicating the relevance of the regulatory effect of the butyrate treatment in bovine cells.

ChIP-seq libraries were obtained from bovine cells treated with butyrate and the control. Butyrate resulted in 23,691,050 clean reads, and the control condition in 23,673,693 clean reads. The Encyclopedia of DNA Elements (ENCODE) project guidelines for transcription factor ChIP-seq recommend at least 20 million usable fragments for each sample (https://www.encodeproject.org/chip-seq/transcription-factor-encode4/, (accessed on 4 April 2022)). According to ENCODE, our samples produced the recommended number of reads; however, at least two replicates per sample are recommended. ENCODE also recommends the fraction of reads in peaks (FRiP score) as a metric to verify the quality of the ChIP-seq studies, and our results showed a FRiP score of 0.22 for butyrate and 0.16 for the control, which indicates an acceptable quality [44].

Of note, 61,915 CTCF peaks were generated for the butyrate condition, representing 1.27% of the cattle genome, and 51,347 for the control, representing 0.88% of the cattle genome (bosTau9). Our results are consistent with a previous study in cattle rumen tissue where the CTCF peaks identified for weaning conditions represented approximately 0.6% of the cattle genome (bosTau8) [14], and a study in sheep that identified CTCF-binding sites representing 0.7% of the sheep genome [45]. We further detected the differentially CTCF-binding sites across the cattle genome by comparing butyrate vs. control conditions. After the detection of the DCBS, filtration steps were performed to ensure its quality, including removing the regions that did not overlap with CTCF peaks and |log_2_FC| < 1. After filtration, we identified 2031 induced DCBS (~90%) and 244 repressed DCBS for the butyrate treatment. We utilized all induced and repressed DCBS for the downstream analysis to identify differences in the biological functions, including annotation, GO, and pathways analyses.

The annotation of the butyrate-induced and -repressed DCBS showed similar results with most of the induced and repressed DCBS in distal intergenic regions, followed by promoters and introns. The induced and repressed DCBS located primarily on distal intergenic regions show a potential role as insulators, as reported in previous studies in sheep [26] and humans [46]. In addition, the distribution of the induced and repressed sites relative to TSS showed that most of the induced and repressed DCBS fall in 10–100 kb, far from the TSS, indicating similar results from a previous study in the vertebrates [47].

GO enrichment revealed important enriched GO terms for the induced DCBS related to cell migration and motility, epithelial cell proliferation, cell adhesion and junction, complex collagen trimers, and growth regulation. We additionally detected a biological relevant KEGG pathway for the induced DCBS, the ECM-receptor interaction. This pathway includes 16 genes such as integrins (*ITGB1*, *ITGA4*, *ITGA5*, *ITGA7*, *ITGB8*), collagens (*COL2A1*, *COL4A1*, *COL4A3*, *COL6A1*), and other genes related to extracellular matrix or cartilage (*COMP*, *FREM1*, *FN1*). The ECM-receptor interaction pathway has a crucial role in maintaining cell and tissue structure and can include collagen, fibronectin, and laminin molecules [48]. The interactions between cells and ECM are mediated mainly by integrins that regulate cell adhesion, migration, proliferation, and differentiation. Integrins have essential roles in regulating cell adhesion, differentiation, and migration [49,50]. In a recent study in cattle, the integrin-linked kinase (ILK)-signaling pathway was identified when analyzing accessible chromatin regions for bovine cells by a butyrate-induced treatment [51]. Collagen fibrils are observed in the rumen epithelium and the core of the rumen papillae [52].

IPA pathway analysis also revealed networks from the induced sites related to cell morphology, assembly, development, growth, proliferation, and signaling, while those from the repressed sites related to cell cycle and movement, and organ development. IPA pathways were obtained for the induced DCBS pertaining to growth hormone, macropinocytosis signaling, regulation of cellular mechanics by calpain protease, and AMPK, and those for the repressed sites related to the regulation of the epithelial–mesenchymal transition, and WNT/β-catenin. Calpains control cell migration by regulating integrin-mediated adhesion and actin-based membrane protrusion [53]. The WNT/β-catenin signaling pathway has roles in regulating cell proliferation, cell determination, and adult tissue homeostasis [54].

Motif enrichment analysis further identified TFBS in the butyrate-induced and -repressed DCBS. CTCF, BORIS, TGIF2, and ZIC3 were identified in the induced and repressed DCBS. As expected, CTCF is enriched for DCBS. BORIS (for brother of the regulator of imprinted sites) or CTCFL is a paralog of CTCF and is involved in methylation events and might be involved in developmental reprogramming and chromatin unfolding [55,56]. TGIFs play a crucial role in energy metabolism regulation in normal cells and can interact with the TGFβ pathway and SMADs [57]. TGIF2 is part of the TALE-homeodomain proteins involved in the cell proliferation and differentiation [58]. The ZIC family proteins play essential roles in vertebrates’ embryonic development [59], and ZIC3 acts by binding the distal regulatory regions and is potentially involved in regulating 300 genes [60]. In addition, ZIC2, ZEB1, and E2A were some of the relevant TFBS identified for the induced sites, while FOS, JUN-AP1, ATF3, FRA1/2, JUNB, and AP1 were some of the TFBS identified for the repressed sites. E2A has roles in cell growth and differentiation and is involved in the transcriptional regulation of several cell lineages [61]. ZEB1 is a crucial transcription factor in the epithelial–mesenchymal transition (EMT) process and is involved in the embryonic development and cancer proliferation [62]. ZIC2 plays an important role during embryonic development and acts as a WNT/β-catenin signaling inhibitor [63,64]. The Activator Protein-1 (AP-1) transcription factors family has several members, including ATF3, JUNB, FRA1/2, and FOS, and they are involved in the cell proliferation and differentiation [65].

DCBS were then integrated with previous RNA-seq data [16] to identify putative target genes. We identified seven target genes for the repressed sites, including *GATA4*, *RASD1*, *KANK3*, and *ZNF395*. Previous studies in mice and quails showed that GATA4 is essential for maintaining intestinal homeostasis and controls functional integrity [66,67] and intestinal epithelial cell proliferation [68]. GATA4 is critical to regulating the intestinal epithelial barrier [69]. Another study in mice showed that oral administration of bovine milk altered gut microbiota and increased the *GATA4* expression in the intestine [70]. Authors also associated SCFAs with the *GATA4* expression, showing that acetate and butyrate positively affected the expression of *GATA4*. RASD1 is part of the Ras superfamily of small GTPases and plays several critical roles, including iron homeostasis, growth hormone secretion, circadian rhythm, and cell proliferation and differentiation [71]. The KANK family plays roles in actin cytoskeleton organization and cell motility; however, KANK3 functions are not well understood, and KANK3 might act as a tumor suppressor [72]. A recent study proposed that *ZNF395* is a novel tumor suppressor gene [73].

Genomic regions were selected for visualization of genes related to potential roles in rumen development during butyrate supplementation, such as cellular adhesion and cell proliferation. Three regions showing CTCF peaks and induced DCBS were selected for four *integrin* genes, including *ITGB1*, *ITGA4*, and *ITGA5* on chromosomes 13, 2, and 5, respectively. This study observed eight induced regions in seven *integrin* genes (*ITGB1*, *ITGB8*, *ITGA4*, *ITGA5*, *ITGA7*, *ITGA10*, and *ITGBL1*). However, no repressed DCBS were in *integrin* genes. As mentioned before, integrins are crucial for cell adhesion and proliferation and might have a role in bovine rumen development, as shown by previous studies in cattle [15,51]. In this study, 10 induced regions were observed in seven *keratin* genes (*KRT7*, *KRT8*, *KRT14*, *KRT83*, *KRT84*, *KRTAP10-2*, and *KRTAP10-8*). Keratins can be found on the rumen surface and result in protection from potential damage [74]. Also, the keratinization of rumen papillae increases when calves are fed a solid diet [75].

## 5. Conclusions

Butyrate utilization can accelerate calves’ gastrointestinal tract development and improve feed efficiency and growth. CTCF is a crucial chromatin organizer and plays an essential role in gene expression regulation. Using the butyrate-induced treatment on bovine cells and the ChIP-seq approach, genome-wide characterization of CTCF-binding sites and differential CTCF-binding sites for the butyrate vs. control comparison was successfully performed here. Then, a total of 2265 differentially CTCF-binding sites comprising ~90% of induced sites were further integrated with gene ontology, IPA and KEGG pathways, motif enrichment, and RNA-seq data to reveal relevant biological roles related to the butyrate-induced condition. Gene ontology enrichment showed crucial GO terms for the induced sites, mainly associated with cellular proliferation, cell adhesion, and growth regulation. Our study revealed candidate genes in bovine cells by butyrate-induced utilization that might be related to the regulation of rumen development such as integrins, keratins, and collagens, and transcription factors such as BORIS, ZIC2/3, GATA4, and TGIF2. However, additional studies with larger sample sizes are needed to confirm these findings.

## Figures and Tables

**Table 1:** CTCF ChIP-seq read statistics showing the number of reads, number and percentages of reads mapped, mitochondrial reads, duplicate reads and reads with mapping quality <10, and the number of clean reads used for peak calling.

Condition	No. of Reads	Mapped Reads (Bostau9)	% of Mapped Reads	MT Reads	% of MT Reads ^1^	Duplicate Reads	% of Duplicate Reads ^1^	MAPQ < 10 Reads	% of MAPQ < 10 Reads ^1^	Clean Reads ^2^
BT	40,990,109	37,691,069	91.95	7089	0.02	6,850,100	18.17	6,715,731	17.82	23,691,050
CT	39,610,540	34,876,230	88.05	6383	0.02	3,774,541	10.82	6,992,633	20.05	23,673,693
Total	80,600,649	72,567,299	-	13,472	-	10,624,641	-	13,708,364	-	47,364,743
Average	40,300,325	36,283,650	90.00	6736	0.02	5,312,321	14.50	6,854,182	18.93	23,682,372

BT: butyrate. CT: control. ^1^ Percentages were calculated considering the total number of mapped reads. ^2^ Reads uniquely mapped, with MAPQ > 10, no duplicate reads or reads located on MT chromosome.

**Table 2:** Peak calling metrics showing the total number of clean reads used to call peaks and calculate the fraction of reads in peaks (FRiP), number of CTCF peaks (FDR < 0.05), number of assigned reads in peaks, FRiP, an average of peak lengths, and the proportion of peaks near TSS (±3Kb, %).

Condition	Clean Reads ^1^	Clean Reads Used for FRiP ^2^	CTCF Peaks ^2^	Assigned Reads in Peaks ^2^	FRiP ^3^	Average Peak Length	Proportion of Peaks Near TSS (±3 Kb, %)
BT	23,691,050	23,055,514	61,915	5,169,204	0.22	509	16.66
CT	23,673,693	23,020,660	51,347	3,742,588	0.16	426	17.95
Total	47,364,743	46,076,174	113,262	8,911,792	-	-	-
Average	23,682,372	23,038,087	56,631	4,455,896	0.19	468	17.31

BT: butyrate. CT: control. ^1^ Reads uniquely mapped, with MAPQ > 10, no duplicate reads or reads located on MT chromosome. ^2^ Reads located on Chromosomes X and unplaced were not included. ^3^ Fraction of reads in peaks.

**Table 3:** Number of butyrate differentially CTCF-binding sites (DCBS) showing the different steps of filtration, including the number of induced and repressed regions.

Filtration Steps of Butyrate DCBS	N	%
Initially detected DCBS (*p*-value < 0.05)	16,532	100
Filtered DCBS (|log_2_FC| > 1)	2355	14.25
Filtered DCBS that overlapped with peaks	2265	13.70
Total of filtered DCBS	2265	100
Induced sites with log_2_FC ≥ 1	2031	89.67
Repressed sites with log_2_FC ≤ −1	234	10.33

## Data Availability

All high-throughput sequencing data analyzed in this study are deposited in NCBI. RNA-seq data are publicly available in the NCBI GEO database under accession number GSE129423 [16]. All CTCF ChIP-seq data were submitted to NCBI, SRA database (BioProject ID: PRJNA672996).

## Supplementary Materials

The following supporting information can be downloaded at: https://www.mdpi.com/article/10.3390/biom12091177/s1, Table S1: Merged peaks information from the butyrate and control conditions and butyrate differentially CTCF-binding sites overlapped with the merged peaks (*p*-value < 0.05 and |log_2_FC| > 1). Table S2. Butyrate-induced and -repressed differentially CTCF-binding sites (DCBS) with annotation information. Table S3. Enriched gene ontology (GO) terms (FDR < 0.05) for genes associated with butyrate-repressed differentially CTCF-binding sites for biological process. Table S4. Enriched gene ontology (GO) terms (FDR < 0.05) for genes associated with butyrate-induced differentially CTCF-binding sites for biological process, cellular component, and molecular function. Table S5. KEGG significant pathways (FDR < 0.05) were obtained from the butyrate-induced differentially CTCF-binding sites. Table S6. Significant networks (network score > 20) were obtained for genes from the butyrate-induced and -repressed differentially CTCF-binding sites (DCBS). Table S7. Significant canonical pathways (*p*-value < 0.01) of genes from the butyrate-induced and -repressed differentially CTCF-binding sites (DCBS). Table S8. Significant upstream regulators (*p*-value of overlap < 0.01) of genes from the butyrate-induced and -repressed differentially CTCF-binding sites (DCBS). Table S9. Enriched motifs (*p*-value ≤ 0.01 and ≥5% of target sequences with motif) were identified in the butyrate-induced and -repressed differentially CTCF-binding sites. Results are based on the HOMER curated motifs database, most of which are based on published Chip-Seq data. Each motif has information about cell type, immunoprecipitated protein, and the Gene Expression Omnibus (GEO) accession number or publication information. Table S10. Downregulated genes targeted by butyrate differentially CTCF-binding sites.

## Abbreviations

**ATAC-seq**	Assay of Transposase Accessible Chromatin sequencing
**BP**	biological process
**BT**	butyrate
**CC**	cellular component
**ChIP-seq**	Chromatin immunoprecipitation followed by sequencing
**CT**	control
**DCBS**	CTCF-binding sites
**ECM**	extracellular matrix
**EMT**	epithelial–mesenchymal transition
**ENCODE**	The Encyclopedia of DNA Elements
**FC**	fold change
**FDR**	false discovery rate
**FRiP**	fraction of reads in peaks
**GEO**	Gene Expression Omnibus
**GO**	Gene Ontology
**IPA**	ingenuity pathway analysis
**MAPQ**	mapping quality
**MDBK**	Madin–Darby bovine kidney
**MF**	molecular function
**MT**	mitochondrial
**SCFAs**	short-chain fatty acids
**TSS**	transcription starting site
